# Keyword Index for Volume 89

**DOI:** 10.1038/sj.bjc.6601556

**Published:** 2003-12-09

*α*-catenin 557

*α*-fetoprotein 1086

*α*-MSH 2004

*α*-tocopheryl succinate 1822

1100delC 1966

^19^F magnetic resonance spectroscopy 754

^19^F MRS 1796

5-aminolaevulinic acid 405, 730

5-fluorouracil 239, 455, 754, 803, 944, 992, 1796, 2213

5-FU 816

72 kDa type IV collagenase 1270

7271T>G mutation 1513

A3 adenosine receptor 1552

abdomino-perineal resection 2019

ablation 2227

abnormal nucleation 1802

accelerated proliferation 2184

acceptability 1400

ACIS 886

actin 860

acute hypoxia 350

adenocarcinoma 834, 2078

adenoviral cytolysis 577

adenovirus 385, 1162, 2140

adjuvant 1837

adjuvant chemotherapy 2062

adjuvant therapy 1042, 1433

adrenomedullin 1927

adriamycin 1423

adult height 81

adult leg length 81

adult trunk length 81

advanced cancer 23

advanced disease 77

advanced gastric cancer 2207

advanced gastrointestinal cancer 2051

advanced melanoma 1901

advanced NSCLC 2190

Africa 65

aggregation state 937

AIDS 94, 505

AKT 185, 391, 2110

ALA efflux 173

ALA uptake 173

albumin 1028

alcohol 1667

allelic loss 2289

alpha-interferon 50

alveolar soft-part sarcoma 243

anal carcinoma 2057

anatomic site 1202

androgen deprivation 971

androgen-independent cell 1566

androgen receptor 552

aneusomy 128

angiogenesis 215, 1375, 1395, 1927

angiopoietins 891

annexin V 1327

anthracyclines 36, 666

anthropometry 519

antiangiogenesis 1395

antiangiogenic 262, 907

antiangiogenic activity 243

antibody–cytokine fusion protein 1130

antibody response 291

antigens 199

antioxidants 1255

antiproliferative 907

antisense oligonucleotides 1352

antitumour effect 1102

AP-2 transcription factor 899

APC 1298

Apo2L/TRAIL 206

apoptosis 178, 192, 206, 363, 564, 727, 763, 915, 944, 1035, 1091, 1108, 1327, 1345, 1358, 1366, 1590, 1620, 1757, 1776, 1802, 1817, 1822, 1896, 1950, 1979, 2155, 2244, 2277

apoptosis resistance 1714

apoptosome 2147

arginine 907

arginine deiminase 907

arginine deprivation 573

arthropometry 81

aspirin 1705

association 1524

astrocytoma grade II 128

ataxia telangiectasia 1091

ATM gene screening 1513

ATP-TCA 2299

autologous transplantation 29

axilla 1310

*β*-catenin 152, 557, 1298

*β*1-integrin 374, 2122

*β*3-integrin 2122

B cell lymphoma 2155

B16-F10.9 melanoma 314

bacteriochlorophyll 2333

Barrett's oesophagus 1508

Bax 1590

BCG immunotherapy 2312

BCL-2 55, 391

Bcl-x_L_ 1352

BCRP/MXR/ABCP/ABCG2 1971

Belarus and Ukrainian registries 2098

benign prostatic hypertrophy 106

best supportive care 2219

betel chewing 1202

betel quid 681

bilateral breast cancer 1513

biomarkers 8, 1940

biopsy 8

birth weight 1664

bispecific antibody 1987

bisphosphonates 178

biweekly schedule 239

bladder cancer 298, 1096, 1290, 2264, 2271, 2312

blindness 2038

blood flow 2333

blood pressure 1243

body mass index 513, 519, 1667

body size 852

bone markers 2031

bone marrow 539

bone metastases 178, 625, 660

BRAF 1958

brain 1743

brain tumour 577

breast 277, 524

breast cancer 15, 77, 81, 135, 178, 185, 192, 284, 305, 492, 508, 533, 539, 648, 666, 840, 847, 977, 983, 1035, 1185, 1255, 1513, 1627, 1645, 1664, 1686, 1693, 1750, 1789, 1920, 1934, 2062, 2289

breast cancer genetic risk counselling 653, 1650

breast cancer in young women 1661

breast cancer risk 1672

breast carcinomas 113, 660, 1502, 2227

breast density 852

breast neoplasms 271, 487

breast tumour 1310

bronchoscopy 1885

brostallicin 1559

brush border 1995

bryostatin-1 1152, 1418

C26 murine colon carcinoma 754

Ca19-9 1413

CA9 877

cachexia 1116

calcitriol 968

caloric restriction 1375

calorie restriction 2254

camptothecin 1757

cancer 120, 1147, 2045

cancer awareness 2165

cancer cachexia 2254

cancer care 821

cancer diagnostics 1067

cancer incidence 1709

cancer of oral cavity and oropharynx 1667

cancer pain 2027

cancer patients 1055

cancer relative survival 74

cancer risk 308

cancer vaccine 1079

canine 1530

capecitabine 615, 1627, 1843

carbonic anhydrase IX 2, 1067, 1290

carboplatin 803, 1901

carcinoid 455

carcinoid tumours 1383

carcinoma 524, 1610

case–control 88, 1255

case–control studies 23, 831, 840, 2087, 1228

caseload 15, 492

caspase-9 1366

caspases 2147

cathepsin 1574

Caveolin 1909

CB1954 944

CD151 158

CD1a 533

CD26/DPPIV 1366

CD4 1736

CD40 ligation 1162

CD8 1736

CD95 363, 1950

CDDP 2207

cDNA array 564

cell cycle 120, 564, 763, 1091, 1920, 2305

cell death 1574, 1590

cell lines 2289

cell surface molecules 1463

cell-cycle 1776

cell-death assay 1789

c-erbB-2 513, 959

cervical cancer 101, 834, 1248

cervical cancer screening model 1830

cervical cytology 88

cervical intraepithelial neoplasia 109

cervix 65

cervix neoplasms 2078

CF101 1552

checkpoint 1966

CHEK2 1966

chemically induced hepatocellular carcinoma 730

chemically induced squamous cell carcinoma 2320

chemoprevention 412, 1705

chemoradiation 1433

chemosensitivity test 1896

chemotherapy 206, 477, 634, 727, 840, 966, 968, 1035, 1192, 1428, 1439, 1517, 1605, 1627, 1849, 2038, 2045, 2057, 2299

child 2038

childhood cancer 1215, 1228

Chinese 1686

*Chlamydia trachomatis* 831

choroid plexus 1743

choroid plexus tumour 1743

chromosome 17 720, 1530

chronotherapy 1870

cigarette smoking 1202

CIN 831

cinical benefit 2207

cisplatin 585, 617, 795, 1192, 1633, 1860, 2045

cisplatinum 1849

citrulline 573, 907

classic Kaposi's sarcoma 1657

clear cell sarcoma 1072

clinical nurse specialist 15

clinical trial 8, 1152

clonotypic PCR 1876

clorgyline 1979

cluster analysis 305

CMF 1837

CML 1162, 1855

c-Myc 1479

Coexisting CIN 886

cognition 971

cohort analysis 1260

cohort study 465, 505, 1221, 1238, 1664, 1709

colitic cancer 1232

collaboration 15

collagen crosslinks 1722

Colo 205 1995

colon 1358

colon cancer 158, 374, 1352, 1439, 2277

colon carcinoma 363, 1552

colorectal adenoma 152

colorectal cancer 23, 465, 477, 612, 707, 1232, 1638, 1870, 1876, 2299

colorectal cancer patients 1400

colorectal carcinoma 146, 992, 1155, 2051

colposcopy 109

combination chemotherapy 795, 992

combination regimens 1789

combretastatin A-4 1334

comet assay 2264, 2271

communication 2069

communication skills training 1445

comparative genomic hybridisation (CGH) 720, 864, 1530

compliance 497

computed tomography scan 482

computer-aided detection 1645

concomitant drugs 1013

concurrent chemoradiation 795

conflict of interest 1405

confocal fluorescence microscopy 1581

consensus method 821

continuous infusional 5-flourouracil 2051

contralateral breast cancer 513

controlled-release morphine 2027

controlled-release oxycodone 2027

core biopsy 1310

cost – benefit analysis 109

cost-effectiveness 634

cost-minimisation 1002

COX-2 1358

CpG island methylation 338

CpG-oligonucleotides 2312

CPT-11 2178

C-reactive protein 612, 1028

CT antigen 291

CTL 1079

culture 539

cumulative prognostic score 1028

cyclin D 1995

cyclin D1 1920

cyclooxygenase-2 1102

*CYP1B1* 1524

CYP3A4 1855

cytochrome *P*450 1855

cytokeratin 539

cytokines 524, 1463

cytological grading 36

cytology 109

cytosine deaminase 1796

cytoskeleton 860

cytostatic 1995

cytotoxic 1166

cytotoxic drugs 2133

cytotoxicity 199

*δ*-aminolevulinic acid 173

D122 Lewis lung carcinoma 314

DAXX 1950

DCE-MRI 1889

DCIS 277, 1920

DcR2 206

death receptors 1714

DEB theory 2254

decision-making 2219

deficiency 573

degradative enzymes 1123

deletion 1729

dendritic cells (DCs) 533, 1162, 1172, 1463, 1736

depletion 1633

deprivation 612, 1650

desmoplastic small round-cell tumour 1159

dextran derivative 215

diagnosis 1645, 1885

diagnostic 2098

diagnostic classification 1599

diet 1255, 1672

differentiation 140, 146

dihydropyrimidine dehydrogenase 816, 1486

dissociation 1817

D-loop 697

DNA damage 333, 1366, 1479, 2271

DNA microarrays 314

DNA minor groove binder 1559

DNA mismatch repair 1559

DNA repair 333

DNA strand breaks 2277

docetaxel 630, 795, 968

dog 1530

domiciliary 2190

dominant negative 1276

dose individualisation 787

dose intensity 2062

dosimetry 840

doxifluridine 1627

doxorubicin 357, 1545, 2140

drug distribution 1581

drug resistance 185, 1581

drug sensitivity 1559

drug-resistance 1971

DSB 593

Du145 593

dysplasia 1232

E2F 120

early breast cancer 1837

early diagnosis 1048

early life risk 81

EBV gene expression 113

E-cadherin 557

ECF 1433

ECOG 1022

economic evaluation 634

effectiveness 88

EGFR 681, 1285, 2327

EGTR 1776

elderly 1428, 1439, 1827

elderly patients 992

ELISPOT assay 1172

endogenous granzyme B 135

endometrial cancer 891, 2023

endometrial carcinoma 697

endometrial hyperplasia 891

endometrial neoplasms 1697

endothelial growth factors 426

energy expenditure 2254

EORTC QLQ-C30 497

EPA 1116

ependyma 1743

ependymoma 1743

epidemiology 94, 101, 1205, 1221, 1243, 1661, 1705

epidermal growth factor receptor 1266

epidermal/mucosal reactions 1102

epidermoid carcinoma A431 cells 215

epirubicin 617, 1192

epithelium 707

Epstein–Barr virus (EBV) 344, 1200

*ERBB2* promoter 899

ET-743 2305

ethnic origin 641

ethnicity 70, 508

EWS/ATF1 1072

*ex vivo* 1789

excimer dye laser 730

expression 1729

extracapsular dissection 1610

extragonadal germ cell tumours 29

facial nerve palsy 1610

FADD 1950

faecal occult blood 23

familial prostate cancer 691

fas ligand 1345

fat 1672, 1686

Fc*γ*RI 2234

febrile neutropenia 43

fermented wheat germ extract 465

FGFR3IIIc 1276

FGFR3IIIS 1276

Fhit 320

fibroblasts 1473

fish 1686

flavones 1255

flavonoids 1255

flow cytometry 1971

fluoropyrimidine 615, 1843

fluoropyrimidine-based chemotherapy 1486

fluorouracil 585, 615, 1155

focal adhesion kinase 140

focused ultrasound surgery 2227

FOLFOX4 455

folinic acid 239

follicular lymphoma 36

follow-up 482

Foscan 398

*FOXO1A* 327

fruits 1209

functional classes 1940

fund raising 2165

*γ*-irradiation 727

G2/M checkpoint 1479

gadopentetate dimeglumine 1889

gallbladder cancer 1736

gastric cancer 676, 1314, 1428, 1433, 2051

gastritis 1314

gastrointestinal neoplasms 426

gastrointestinal stromal tumours 460

G-CSF 2234

*gef* gene 192

gefitinib 1827

gelatinase A 1270

gemcitabine 239, 1180, 1192, 1413, 1865, 2190, 2299

gene amplification 552

gene expression 314, 564, 1508, 1914

gene expression profiling 1599

gene therapy 192, 2155

genetic testing 1400

Germany 1205

germ-cell tumours 787, 1849, 2202

gestational trophoblastic disease 2197

Gleason score 106

Gleevec 1855

glioblastoma 1896, 2122

glioma 248, 727, 922, 1172, 1375, 1896

Glivec 634, 1403, 1855

glucose transporter protein 1 (GLUT1) 1290

GLUT-1 870

glutathione 1108

GM-CSF 1162

grade 1096

graded-field gel electrophoresis 593

growth 852

growth factor 178

growth hormone 524, 1108

growth pattern 1920

GSK3*β* 1298

GSTM1 1502

GSTT1 1502

HAART 457

haematological 1389

haemoglobin 977

haemolymphopoietic neoplasms 94

haemolysis 1334

haplotype 1524

HCT-15 1995

head and neck cancer 585

head and neck squamous cell carcinoma 864

health economics 1405

heat shock cognate protein 70 1079

HeLa 593

*Helicobacter pylori* 1314

hepatectomy 1493

hepatic artery chemoembolisation 1423

hepatic neoplasms 1423, 1493

hepatocellufalar carcinoma 1614

hepatocellular carcinoma 291, 730, 1086, 1865

hepatocyte culture 1493

hepatocyte injury 1423

Her-2 681

hereditary 1524

hereditary nonpolyposis colorectal cancer 1400

HER-2/*neu* 666, 983, 2110

HER-2/neu 959, 2234

HER-2/neu peptides 1055

*HER2/neu* 1305

HIF 877

high-dose carboplatin 787

high-dose chemotherapy 29, 787, 1159

high-grade cervical dysplasia 1062

high grade intraepithelial lesions 101

high-intensity focused ultrasound 2227

histology 109

HIV 457, 502, 505

HLA-A26 1055

HLA-A3 1055

HLA-B46 1079

HMGA2 2104

hnRNP K protein 1493

Hodgkin's disease 482

hormone replacement therapy 1697

hormone-resistant prostate cancer 552

hormones 1147

hPif1 713

HPV 831, 1248

HPV genotypes 886

HRT 277

HSP27 1950

HSV-TK 1086

HT-29 1995

hTERT 922, 1473

human 899

human catalase 2140

human glioma cell line 1802

human mammary epithelial cells 1479

human monoclonal antibody 1545

Human papillomavirus 101

human papillomavirus 1830

human papilloma virus load 109

human papillomavirus-16 672

Hurthle cell carcinoma 258

HUVECs 357

hyperbilirubinaemia 1403

hypermethylation 1473

Hypersensitivity reaction 477

hyperthermia 405, 2333

hypomagnesaemia 1633

hypopharynx 1940

hypoxia 271, 870, 1290, 2133

hypoxia-inducible factor 2

hypoxia-inducible-factor 1*α* 1042

I157T 1966

IAP 2147

ICBP90 120

IGF-1 1375

IL-12 1552

IL-2 1876

IL-6 338

imatinib 460, 1403, 1855

immortalization 2293

immune privilege 1345

immunochemical test 23

immunocytochemistry 899

immunocytokine 1130

immunohistochemistry 140, 320, 546, 1042, 1729, 1736, 1927, 2249

immunoliposomes 1545

immunostaining 2104

immunotherapy 385, 1130, 1147, 1162, 1172, 2213, 2234

imprint cytology of biopsied sample 1885

incidence 70, 834, 1205

induction chemotherapy 2184

infants 1605

infertility 1849

infrared-A-radiation 405

*In situ* hybridisation 128, 891

insulin 1697

insulin-like growth factor binding protein-1 (IGFBP-1) 1697

insulin-like growth factor binding protein-3 (IGFBP-3) 1697

insulin-like growth factor-I (IGF-I) 1697

interferon alpha 2213

interferon alpha-2b 243

interleukin-2 50, 1620, 2213

interstitial fluid pressure 1334

intestinal metaplasia 1314

intralesional therapy 1620

intramuscular administration 1228

invasion 1123, 1270, 2122

invasive lymphocytes 113

*in vitro* 2133

ionising radiation 1102, 1479, 1802

ionizing radiation 1709

‘Iressa’ 585

Irinotecan 617, 630, 992, 997, 1008, 1439, 1860, 1870

Israeli Jews 1657

IVS10-6T>G mutation 1513

JAK/STAT pathway 338

Japanese population 691

jaundice 1403

JCOG 2178

JNK 1950

KAI1/CD82 158

Kaplan-Meier 232

Kaposi's sarcoma 502

keratinocytes 1473

ketogenic diet 1375

Ki-67 index 128

kidney cancer 1418

kidney tumour 1285

*KIT* gene 460

knockout mice 1776

KSHV 1657

KSHV/HHV-8 502

lactate dehydrogenase 877

language of consultation 641

laryngeal brushing 1048

larynx 1048

laser scanning cytometry 8

*l*-deprenyl 1979

leadership 15

leanness 1667

leukaemia 1228, 1638

lichen sclerosus 2249

life-course 852

LIG-1 1285

linkage 505

lipiodol 1423, 1614

liposarcoma 1409

liposomal doxorubicin 1180

liver metastases 284

*LKB1/STK11* 308

LNCaP 593

local tumour control 2019

loss of heterozygosity 333, 2289

low-dose methotrexate 2197

low-risk 43

lung cancers 320, 702, 795, 959, 1013, 1022, 1705

LVT 930

lymphangiogenesis 426

lymphatic system 426

lymph node metastasis 1042, 1750, 2116

lymphoma 1530, 2087

lysosomes 1574

magnesium 1633

malignancies 1389

malignant effusion 1876

malignant fibrous histiocytoma 720

malignant transformation 2293

mammary cancer 385

mammographic parenchymal patterns 852

mammography 1645

MAPK 185

marine natural compounds 763

matrix metalloproteinase-2 2122

matrix metalloproteinase-7 2116

matrix metalloproteinase-9 2116

MBD2 1934

MCF-7 593

*MC1R* 1961

MDX-H210 2234

MeCP2 1934

mediastinal primary 29

megakaryocytic differentiation 1320

mEH 702

melanocortin 2004

melanocytes 1072

melanoma 573, 1123, 1517, 2004

melanoma-follow-up 1457

melanoma-prognosis 1457

men 1243

*MET* 327

meta-analysis 55, 101, 1672

meta-iodobenzylguanidine 1383

metabolic control 1375

metabolic status 2333

metabolism 573

metastasis 314, 557, 1270, 1909, 2004

metastatic bone disease 2031

metastatic colorectal cancer 1486

metastatic gastric cancer 997

methyl-CpG-binding protein 1934

MIBG 1383

microarray 1599, 1757, 1914

microenvironment 2

micrometastasis 676

micronuclei 727

microsatellite instability 707

midkine 1086

migrants 508

migration 1123

minichromosome maintenance protein-2 1048

missing data 781

MITF 1072

mitochondrial DNA 697

mitochondrial microsatellite instability 697

mitogen-activated protein kinase (MAPK) 1783

mitomycin C 2051, 2299

mitoxantrone 1971

MMP 1817

MMP-2 1123

molecular epidemiology 702

molecular mechanisms 585

monoamine oxidase inhibitors (MAOIs) 1979

MonoHER 357, 2140

morbidity 648

morphology 1693

mortality 77

mouse 1796

mouth 2244

MRP-1/CD9 158

MTC 2093

m-THPC 398

MTT 357, 2140

MUC1 1130

multidisciplinary team 15

multivariate analysis 1166

mutations 308, 1729, 1958, 1971, 2249

myeloprotection 1552

myogenesis 327

n-3 fatty acids 1686

n-3 polyunsaturated fatty acids 1102

n-6 fatty acids 1686

N-telopeptide 2031

Na^+^/H^+^ antiporter 1395

nasopharyngeal carcinoma (NPC) 344

natural killer cells 1130, 1552

natural killer cells (NKCs) 1736

natural regression 1062

NE tumours 1776

neoadjuvant chemotherapy 1185

neonatal cardiac myocytes 2140

neoplasms 1243, 1260

neoplastic disease 1822

neuroblastoma 412, 470, 860, 1605

neuroendocrine tumour 455

neuropsychology 2038

neutropenia 1837, 2062

neutrophil adhesion 357

neutrophils 1345

NF-kappaB 2004

NF-*κ*B 391, 1108, 1116, 1358

NICE 966

nitroreductase 944

NO 907

node-positive breast cancer 268

noncytotoxic 1166

non-Hodgkin 2087

non-Hodgkin lymphoma 713

non-Hodgkin's lymphoma 1958

nonimmune cells 135

nonseminomatous histology 29

non-small-cell lung cancer 55, 246, 457, 877, 1008, 1028, 1192, 1789

normal cells 1147

normal ovary 1298

normoxia 2133

Norway 1238

Nottingham prognostic index 1185

NSAIDs 1358

NSCLC 1827, 2184

Ntx 2031

nucleophosmin/B23 1320

nude mice 1383

obesity 519

occupational exposures 1215

octreotide 258

oesophageal adenocacenoma 1508

oesophageal adenocarcinoma 1729

oesophageal cancer 140, 630, 1202, 1209, 2299

oesophageal squamous cell carcinoma 1042

oesophagus 2045

oestrogen and progesterone receptor status 1661

oestrogen receptor 113, 1934

OK-432 1876

oligodendroglioma 2327

*O*^6^-methylguanine-DNA-methyltransferase 1517

oncogenes 1305, 1958

oncogenesis 1395

oncologist 1022

oncology 1405

optic pathway glioma 2038

oral 808, 1627

oral antibiotics 43

oral cancer 681

oral mucosa 2244

oral squamous cell carcinoma 557, 1722

organ transplantation 1221

orotate phosphoribosyl transferase 1486

osteosarcoma 206

OT 930

outcome and process assessment (health care) 15

outpatient 43

ovarian cancer 966, 1002, 1152, 1180, 1298, 1843, 2293

ovarian malignancy 672

ovarian neoplasm 1002

ovarian surface epithelium 2293

overexpression 1574

oxaliplatin 1155, 1439, 1870

oxygenation 2333

oxygen consumption 937

oxygen tension 350

oxytocin receptor 930

p16 1802

p185 959

p21 ^WAF1/CIP1^induction 1566

p21 ^WAF1/CIPI^-deficit cell 1566

p21 ^Waf1/Cip1^ 1757

p31 EBV DNA 113

p38MAPK 564

p53 320, 707, 727, 1314, 1729, 2249

p53 tumour suppressor gene 672

paediatric Hodgkin's lymphoma 1200

palladium-bacteriopheophorbide 2320

palliative care 2069

palliative chemotherapy 1860, 2219

pancreas cancer 391, 1987

Pancreatic adenocarcinoma 239, 1714

pancreatic cancer 1413, 1860, 2104, 2110

pancreatic ductal adenocarcinoma 338

pancreatic neoplasms 519

papillomavirus 94

Pap smears 88

parotid gland 1610

patient-doctor communication 2202

patient participation 2069

patient preferences 1450

patient selection 1860

*PAX3* 327

*PAX7* 327

PCR 1750

pegylated liposomal doxorubicin hydrochloride 1002

performance status 1022, 1028

period analysis 1260

period survival 74

peripheral benzodiazepine receptor 564

peripheral lung cancer 1885

peritoneal cytology 2023

PET 8

PET imaging 1327

Peutz–Jeghers syndrome 308

P-glycoprotein 1581

pH 2

pharmaceutical targets 1940

pharmacodynamics, hamster tumour model 2320

pharmacokinetics 398, 816, 1776, 1822, 1855

phase I study 617, 1901

phase I/II study 2051

phase II 997, 1428

phase II study 1008

phase II trial 1418

phase III trial 1192

phase one 1166

phenylacetate 412

phenytoin 615

pheochromocytoma 1383

phosphatidylserine 1327

phospholipase A_2_ (PLA_2_) 1783

phospholipase C (PLC) 1783

photodynamic diagnosis 730

photodynamic therapy 173, 398, 405, 937, 1590, 2320, 2333

photodynamic therapy (PDT) 730

photosensitiser 937, 2320

pH regulation 1395

phthalocyanine Pc 4 1590

physical activity 847

physician patient relations 2069

pimonidazole 1290

plasminogen activation and metastasis 374

platinum compound 477

*p*O_2_ fluctuations 350

point mutations 687

polyethylene glycol 937

polyethyleneglycol 1545

polylysine conjugate 937

polymorphisms 702, 1096, 1502, 1517

polyomavirus 385

polypharmacotherapy 1013

polyunsaturated fatty acids 1686

pooled analysis 1638

poor PS patients 1827

population pharmacokinetics 787

positron emission tomography 262

potassium 1633

PPAR*γ* 1409

preclinical models 2327

preconception exposure 1709

predictive assay 2277

predictive factors 977

predictive test 2264

predictors 1062, 1248

preterm birth 1664

prevention 2165

primary care 1650

primary chemotherapy 666, 977

primary language 641

procarbazine 248

prognosis 128, 140, 271, 298, 460, 470, 660, 681, 720, 1022, 1166, 1260, 1266, 1450, 1502, 2045

prognostic classification 1599

prognostic factors 546, 959, 2116

prognostic indices 1909

prognostic variables 284

progression-free and overall survival 465

prolactin 524

proliferation 128, 1035, 1493, 1817

proliferation index 2093

promoter 1086

prospective cohort study 847

prospective studies 1705

prostate cancer 106, 625, 968, 971, 1055, 1238, 1524, 1966

prostate tumours 687

proteasome expression 1116, 1783

protein catabolism 1116

protein kinase C 1152, 1418

proteolysis-inducing factor 1116

proteolysis-inducing factor (PIF) 1783

proteome 305

psychological impact 653

pyridinoline 1722

quality of life 50, 497, 641, 648, 971, 1843, 2202

quantitative real-time RT–PCR 271

quantitative RT– 1750

question prompt list 2069

radiation 593, 1215, 2305

radiation enhancement 1987

radiation protection 1979

radioimmunoassay 1927

radioiodine 258, 1638

radioligand 930

radiosensitisation 1352, 2305

radiosensitivity 2264, 2271

radiosensitization 577

radiosensitizer 746

radiotherapy 65, 298, 840, 2023, 2038, 2057, 2271

Raman spectroscopy 106

RARRES3 146

RAS 185, 1958

rat sarcoma 405

razoxane 262

RCAS1 546

RCC 1285

reactive oxygen species 1314

recombinant bacteria 1796

rectal cancer 870

recurrence 277, 1920

registries 1205, 1260

relapse 482

renal cell carcinoma 50, 1266, 1909, 2147, 2213

repeated events 781

residual disease 1185

resistance 206, 1147

response 277

retinoblastoma protein 120, 298

retinoblastoma tumour suppressor gene 135

retinoid 808

retinoyl glucuronide 412

reverse transcription–polymerase chain reaction 676

rhabdomyosarcoma 327

rhenium-188-HEDP 625

RhG-CSF 1008

RIG 1 146

rinse-fluid cytology of forceps and brush 1885

risk factors 519, 831, 2078

rituximab 1389

*RNASEL* gene 691

RNF11 1538

rosiglitazone 1409

RT112 593

RT–PCR 1457, 2104

rural health services 821

S-1 816, 2207

salted meat 1209

sarcoma 720

scFv 1130

Scotland 505

screening 23, 77, 1645

screening interval 88

seasonality 1200

second cancer 840

second-line treatment 2213

second primary malignancies 1638

senescence 1473

sentinel lymph node 1750

sentinel lymph node biopsy 648

sentinel node 676

seroprevalence 1248

serrated adenoma 152

serrated polyp 152

service delivery 653, 1650

sex steroids 385

sexual functioning 2202

side effects 1633

signal transducers and activators of transcription (STAT) 344

simvastatin 1855

skeletal-related events 2031

skin neoplasms 1205

SM-11355 1614

Small cell lung cancer 55, 2178

smoking 831, 1667, 2087

Smurf2 1538

socioeconomic status 1693

SOCS 524

*SOCS-1* 338

soft-tissue melanoma metastases 1620

soluble protein 1276

somatostatin receptor 258

South Asians 508

southern blot assay 676

SPARC molecular markers 1508

splice variant 1276

sponsorship 1405

squamous cell carcinoma 101, 834, 1048, 2078, 2244

squamous cell carcinoma of the vulva 2249

squamous dysplasia 1048

stage 1028, 1693

stage 4s 470

stage III 1457

stamps 2165

statistical methods 232

stem cells 1776

steroid receptors 268

stewed meat 1209

streptozocin 455

stroma 707

stroma-supported immunocytometric assay 763

superficial oesophageal cancer 2116

supportive care 1013

suppressor genes 687

surgery 460, 487

surveillance colonoscopy 1232

survival 55, 65, 487, 508, 533, 612, 660, 834, 864, 959, 1260, 1610, 1693, 1837, 1896, 2023, 2045, 2093

survival analysis 232, 271, 781

survivin 1743, 2244

susceptibility 1502

synergistic 746

synergy 944

systematic review 55, 959

T47D 593

tamoxifen 268, 277

tankyrase 713

targeting 1545

tazarotenic acid 808

T-cell precursor frequencies 1055

T-cell receptor (TCR) 1876

Tcf/Lef 1298

TCL1 1091

telomerase 713, 922

telomere 1091

temoporfin 398

temozolamide 248

temozolomide 922, 1901

temporal heterogeneity in *p*O_2_ 350

testicular cancer 2202

testicular germ cell tumours (TGCTs) 2133

Tet system 2155

TGCT cytokine 915

TGF*β* signalling 1538

therapeutic efficiency 625

therapy 1147, 1358, 2227

the systemic inflammatory response 612

thoracic radiation therapy 803

thyroid cancer 1638

thyroid carcinoma 2098

TIG3 146

TIMP 1817

tissue 1147

tissue samples 1722

TK 1776

TM4SF 158

TNF 1096

TNF-*α* 1123

toll-like receptor 2312

Tookad® 2320

topoisomerase II 1366

topoisomerase IIα 120

Topoisomerase II*α* 666

topotecan 1002, 1789

toxicity 2057

TPA 1320

TRAIL 363, 2155

TRAIL receptors 363

trans-arterial chemoembolisation 1614

transforming growth factor-*β* 1345

transitional cell carcinoma 1305

trastuzumab 983

treatment 65, 470, 492

treatment choice 2219

treatment preference 2219

TRF1 713

TRF2 713

tropomyosin 860

truth disclosure 2069

tumorigenesis 1817

tumour antigens 1067, 1130

tumour biology 1508

tumour blood flow 1334

tumour doubling time 2184, 2254

tumour epitopes 1079

tumour growth rate 2254

tumour immunity 199, 1463

tumour marker 1927

tumour models 1581

tumour necrosis factor alpha 1987

tumour perfusion 262

tumour response 977

tumours 593, 930, 1276

tumour suppressor gene 146

tumour thickness 2019

tumour vessel 1334

TUNEL 1327

two-dimensional electrophoresis 305

tyrosinase 1457

tyrosine kinase 1783

tyrosine kinase inhibitor 246, 1889

tyrosine kinase phosphorylation 983

ubiquitination 1538

Uganda 502

ulcerative colitis 1232

ultrasound 1310

understanding 1450

understanding of disease status 641

‘unknown’ genes 1940

unresectable 1605

upper gastrointestinal cancer 497

urokinase plasminogen activator receptor 374

uterine endometrial cancer 546

uveal melanoma 1914, 1961

vaccination 199

validation 781

vascular endothelial growth factor 268

vascular endothelial growth factor (VEGF) 215

vascular invasion 1909

vascular permeability 1889

VDEPT 944

vegetables 1209

VEGF 1889

VEGF-B 891

vesicular monoamine transporter 1383

vinorelbine and paclitaxel 1566

virtual Northern 1940

visual acuity 2038

vitamin D 746, 968

vitamin E analogues 1822

vitamin K 1228

VMAT 1383

waiting times 492, 2184

weekly 1428

whitehall 1243

WHO classification 36

wide local excision 2019

WISP-1 314

Wnt-signalling 1298

Wnt signal transduction 152

women 2087

workload 15, 487

written information 1450

xenograft human tumour 1796

xenografts 577, 2289, 2327

xeroderma pigmentosum 333

young women 88

ZD 1839 246, 585

ZD6474 1889

zemplar 746

